# Neurogenin 3 mediates sex chromosome effects on the generation of sex differences in hypothalamic neuronal development

**DOI:** 10.3389/fncel.2014.00188

**Published:** 2014-07-08

**Authors:** María J. Scerbo, Alejandra Freire-Regatillo, Carla D. Cisternas, Mabel Brunotto, Maria A. Arevalo, Luis M. Garcia-Segura, María J. Cambiasso

**Affiliations:** ^1^Laboratory of Neurophysiology, Instituto de Investigación Médica Mercedes y Martín Ferreyra, INIMEC-CONICET – Universidad Nacional de CórdobaCórdoba, Argentina; ^2^Instituto Cajal, Consejo Superior de Investigaciones CientíficasMadrid, Spain; ^3^Departamento de Biología Bucal, Facultad de Odontología – Universidad Nacional de CórdobaCórdoba, Argentina

**Keywords:** Ngn3, sex differences, sex chromosomes, estradiol, neuritogenesis, hypothalamic neurons

## Abstract

The organizational action of testosterone during critical periods of development is the cause of numerous sex differences in the brain. However, sex differences in neuritogenesis have been detected in primary neuronal hypothalamic cultures prepared before the peak of testosterone production by fetal testis. In the present study we assessed the hypothesis of that cell-autonomous action of sex chromosomes can differentially regulate the expression of the neuritogenic gene neurogenin 3 (Ngn3) in male and female hypothalamic neurons, generating sex differences in neuronal development. Neuronal cultures were prepared from male and female E14 mouse hypothalami, before the fetal peak of testosterone. Female neurons showed enhanced neuritogenesis and higher expression of Ngn3 than male neurons. The silencing of Ngn3 abolished sex differences in neuritogenesis, decreasing the differentiation of female neurons. The sex difference in Ngn3 expression was determined by sex chromosomes, as demonstrated using the four core genotypes mouse model, in which a spontaneous deletion of the testis-determining gene Sry from the Y chromosome was combined with the insertion of the Sry gene onto an autosome. In addition, the expression of Ngn3, which is also known to mediate the neuritogenic actions of estradiol, was increased in the cultures treated with the hormone, but only in those from male embryos. Furthermore, the hormone reversed the sex differences in neuritogenesis promoting the differentiation of male neurons. These findings indicate that Ngn3 mediates both cell-autonomous actions of sex chromosomes and hormonal effects on neuritogenesis.

## INTRODUCTION

Since the pioneering work of Phoenix et al. (1959), showing that testosterone during the fetal period masculinizes and defeminizes sex behavior in the female guinea pig, numerous laboratories have identified the organizational action of testosterone during critical periods of development as the main cause of the generation of sex differences in the brain (Arnold and Gorski, 1984). This action of testosterone is in part mediated by its local conversion into estradiol by the enzyme aromatase (MacLusky and Naftolin, 1981). The developmental actions of estradiol in the brain have been well characterized, especially in the hypothalamus where the hormone regulates neuritogenesis and generates sex differences in neuronal circuits controlling neuroendocrine events, feeding, growth, and reproduction (Ferreira and Caceres, 1991; Díaz et al., 1992; Lenz and McCarthy, 2010; Semaan and Kauffman, 2010).

Not all sex differences in the nervous system can be attributed to the actions of gonadal hormones. For instance, sex differences have been detected in the development of neurons and glial cells in mesencephalic and hypothalamic cultures obtained from rat and mouse embryos at embryonic day 14 (E14; Engele et al., 1989; Reisert et al., 1989; Beyer et al., 1990, 1992; Reisert and Pilgrim, 1991) and E16 (Cambiasso et al., 1995, 2000), respectively. These sex differences cannot be attributed to the peak of testosterone production by fetal testis, which in mice is at E17-18 (O’Shaughnessy et al., 1998, 2006) and in rats at E18.5-19.5 (Huhtaniemi, 1994; Scott et al., 2009). However, it is not possible to completely exclude any effect of hormones derived from the gonads at or before the embryonic age used.

Recent studies have explored whether cell-autonomous actions of sex chromosomes (i.e., actions that are not mediated by the hormones produced by the gonads) are involved in the generation of sex differences in the nervous system. This has been facilitated by the development of the four core genotypes mouse model, in which a spontaneous deletion of the testis-determining gene Sry from the Y chromosome (Y^-^) was combined with the insertion of the Sry gene onto an autosome (Lovell-Badge and Robertson, 1990; Mahadevaiah et al., 1998). Mating XY^-^Sry males to XX females produces four type of progeny: XX females, XY^-^ females, XXSry males and XY^-^Sry males. These males are masculinized equivalently by testosterone secretions during development, and thus differ phenotypically from both female groups (De Vries et al., 2002; Arnold and Burgoyne, 2004; Arnold and Chen, 2009). Cell-autonomous actions of sex chromosomes on sex differences in the number of dopaminergic neurons in primary mesencephalic cultures (Carruth et al., 2002), in the density of vasopressin immunoreactive fibers in the lateral septum (De Vries et al., 2002), in nociception (Gioiosa et al., 2008), in some aspects of social behavior (Gatewood et al., 2006), in habit formation (Quinn et al., 2007), in motor deficits in experimental autoimmune encephalomyelitis (Smith-Bouvier et al., 2008), in Ang II-bradycardic baroreflex response (Caeiro et al., 2011), and in sodium depletion-induced brain activity (Dadam et al., 2014) have been identified using this model.

In the present study we have tested the hypothesis of cell-autonomous sex-biasing action of X and Y genes (Davies et al., 2006; Arnold and Chen, 2009; Kopsida et al., 2009; Arnold et al., 2013) can differentially regulate the expression of neuritogenic genes in male and female hypothalamic neurons, generating sex differences in neuronal development. In particular, we have focused on the autosomal gene neurogenin 3 (Ngn3; MGI:893591; located in chromosome 10 in mouse and humans), which is a gene required for the correct specification of neuronal subtypes controlling energy homeostasis in the ventromedial hypothalamus (Pelling et al., 2011) and has recently been implicated in neuritogenesis (Salama-Cohen et al., 2006; Simon-Areces et al., 2010). In addition, recent studies have shown that Ngn3 expression is upregulated by estradiol in hippocampal neurons (Ruiz-Palmero et al., 2011). Since during the critical period of hormonal-induced brain sex differentiation estradiol, produced in the male brain from peripheral testosterone, is involved in the generation of sex differences in hypothalamic neurons (Matsumoto and Arai, 1986), we have also investigated the effect of the hormone on neuritogenesis and Ngn3 expression.

Our findings indicate that the differential expression of Ngn3 in male and female neurons, which is regulated by sex chromosomes, is involved in the generation of sex differences in the rate of neuronal differentiation. In addition, estradiol enhances the differentiation of male neurons through increasing Ngn3 levels, abolishing sex differences in neuronal development. These findings indicate that sex chromosomes regulate sex differences in the rate of hypothalamic neuronal development by an interaction of direct cell autonomous actions and indirect actions mediated by gonadal hormones. Therefore, the process of neuronal sex differentiation seems to be more complex than previously thought.

## MATERIALS AND METHODS

### ANIMALS

The embryos used for this study were obtained from CD1 mice raised in the Cajal Institute (Madrid, Spain) and MF1 four core genotypes (FCG) mice born and reared in the Ferreyra Institute (Córdoba, Argentina). The day of vaginal plug was defined as E0. All procedures for handling and killing the animals used in this study were in accordance with the European Commission guidelines (86/609/CEE and 2010/63/UE) and the Spanish Government Directive (R.D. 1201/2005). Experimental procedures were approved by the Cajal Institute Ethic Committee of Animal Experimentation.

The FCG mouse model combines a deletion of the testis-determining gene Sry from the Y chromosome (Y^-^) with the subsequent insertion of a Sry transgene onto an autosome (Lovell-Badge and Robertson, 1990; Mahadevaiah et al., 1998). The Sry gene deletion in XY mice (XY^-^) yields in a female phenotype (ovaries). When the Sry transgene is inserted into an autosome of these mice they have testes and are fully fertile (XY^-^*Sry)*. Male and female are defined here according to the gonadal phenotype. The Y^-^ chromosome and the Sry transgene segregates independently, thus, four types of offspring are produced by breeding XY^-^*Sry* males to XX females: XX and XY^-^ females (without Sry on the Y chromosome) and XX*Sry* and XY^-^*Sry* male mice (both with *Sry* in an autosome). By comparing these genotypes, it is feasible to segregate the role of (a) sex chromosome complement (comparing mice with the same gonadal type but with different sex chromosomes: XX vs. XY) (b) gonadal phenotype (males vs. females regardless of the sex chromosome complement) and (c) their interaction (Arnold and Chen, 2009). Throughout the text, we will refer to XX and XY^-^ as XX and XY females, and to XXSry and XY^-^Sry as XX and XY male mice, respectively.

### HYPOTHALAMIC NEURONAL CULTURES AND CELL TREATMENTS

Hypothalamic neurons were obtained from embryonic day 14 (E14) mouse embryos. Cells were cultured separately according to the sex and/or genotype of fetal donors. Male fetuses were identified under a dissecting microscope by the presence of the spermatic artery on the developing gonad. The brain was dissected out and the meninges were removed. Then, the ventromedial hypothalamic region, delimited by the optic chiasm, the lateral hypothalamic sulcus and the mammillary bodies, was dissected out from the diencephalon. The blocks of tissue were dissociated to single cells after digestion for 15 min at 37°C with 0.5% trypsin (Worthington Biochemicals, Freehold, NJ, USA) and DNase I (Sigma—Aldrich Co., St. Louis, MO, USA) and washed in Ca^2+^/Mg^2+^-free Hank’s Buffered Salt Solution. Neurons were cultured in phenol red-free Neurobasal supplemented with B-27 and GlutaMAX I (Invitrogen, Crewe, UK). For morphometric analysis, cells were plated on glass coverslips at a density of 150 cells/mm^2^ and maintained *in vitro* for 1–7 days. Some cultures were treated for 4 days *in vitro* (DIV) with 17β-estradiol (10^-10^ M; Sigma-Aldrich) or vehicle. For gene expression analyses, cells were plated on 6-wells plates at a density of 800 neurons/mm^2^ and after 3 DIV the medium was replaced for 2 h by fresh medium devoid of B27 and GlutaMAX I supplement. Then, some cultures were incubated for 2 h with 17β-estradiol (10^-10^ M) or vehicle. The surfaces of glass coverslips and plates were coated with poly-L-lysine (Sigma-Aldrich).

### GENOTYPING

Genotyping of FCG was performed on genomic DNA samples of E14 mouse embryos by PCR for the *Sry* transgene [primers SryF (forward): CTA CAC AGA GAG AAA TAC CCA AAC; SryR (reverse): GTC TTG CCT GTA TGT GAT GG] (Gubbay et al., 1990) and the Y long-arm gene family Ssty [primers SstyF (forward): CTG GAG CTC TAC AGT GAT GA; SstyR (reverse): CAG TTA CCA ATC AAC ACA TCA C] (Turner et al., 2000). The autosomal gene myogenin [primers MYOF (forward): TTA CGT CCA TCG TGG ACA GCA T; MYOR (reverse): TGG GCT GGG TGT TAG TCT TAT] served as an amplification control (Palaszynski et al., 2005) yielding a 245-bp product in all genotypes. Amplification of DNA yielded the following products according to the genotypes: for XY males the 159-bp Sry and the 302-bp Ssty; for XY females the 302-bp Ssty; for XX males the 159-bp Sry; meanwhile in XX females only the myogenin control product was amplified.

### INHIBITION OF Ngn3 EXPRESSION USING SMALL INTERFERING RNAs

Small interfering RNA (siRNA) oligonucleotides were purchased from Applied Biosystems (Weiterstadt, Germany)/Ambion (Austin, TX, USA) and the concentration was 25 nM during transfection. A siRNA targeting Ngn3 (sense, AACUACAUCUGGGCACUGAtt; antisense, UCAGUGCCCAGAUGUAGUUgt) and a control non-targeting siRNA were used. The efficacy of these siRNAs was previously demonstrated (Ruiz-Palmero et al., 2011). For morphological analysis neurons were co-transfected at 3 DIV with pEGFP-C2 (Clontech, USA) plus one of the siRNA oligonucleotides, using the Effectene Transfection Reagent (Qiagen GmbH, Hilden, Germany), following the manufacturer’s instructions. After 16 h of treatment the cultures were processed for immunostaining. The transfection efficiency was 0.1%. Transfected neurons were recognized by EGFP expression. For biochemical analysis, the same plasmids and siRNAs were nucleofected into cultured neurons using an Amaxa nucleofector with the Mouse Neurone Kit (Amaxa, Gaithersburg, MD, USA) in accordance with the manufacturer’s instructions. After 1 DIV, neurons were harvested and processed for real time PCR analysis. The transfection efficiency with this method was 90%. For data representation, we did not take in consideration the 10% of cells that were not nucleofected.

### ANALYSIS OF GENE EXPRESSION BY QUANTITATIVE REAL-TIME POLYMERASE CHAIN REACTION (q-PCR)

Total RNA was extracted from cultures with illustra RNAspin Mini RNA isolation kit from GE Healthcare (Buckinghamshire, UK). RNA was eluted in 100 μl of RNase-free water and absorbance was measured at 260 nm to determine concentrations. cDNA was synthesized from 2 μg of total RNA by using the First Strand cDNA Synthesis Kit from Fermentas GMBH (St Leon-Rot, Germany) following the manufacturer’s instructions. Quantitative real-time PCR was performed using the ABI Prism 7000 Sequence Detector (Applied Biosystems, Weiterstadt, Germany), with conventional AB cycling parameters (40 cycles of 95°C, 15 s; 60°C, 1 min). TaqMan probes and primers for Ngn3 and for the control housekeeping gene, Gapdh, were Assay-on-Demand gene expression products (Applied Biosystems). Real-time PCRs were performed following the suppliers instructions using the TaqMan PCR Master Mix. All reactions were done in triplicates, from six different cultures. Ngn3 expression was normalized for Gapdh expression. To determine the effect of the experimental treatments on the expression of the internal control gene, the relative amounts of Gapdh were calculated using 2^-ΔT^ equation where ΔCT = CT treated – CT control (Livak and Schmittgen, 2001). No significant relationships were found between the treatments and expression of Gapdh (*p* > 0.05) in any of the experiments done. Relative mRNA expression level was calculated using the ΔΔCT method.

### IMMUNOCYTOCHEMISTRY

After 1–7 DIV or after treatment with siRNAs, cells were fixed for 20 min at room temperature in 4% paraformaldehyde and permeabilized for 4 min with 0.12% Triton-X plus 0.12% gelatin in phosphate buffered saline (PBS). Cells were then washed with PBS/gelatin and incubated for 1 h with anti-microtubule associated protein-2 (MAP-2) mouse polyclonal antibody (diluted 1:250 in PBS/gelatin; Sigma-Aldrich), with anti-Tau rabbit monoclonal antibody (diluted 1:250 in PBS/gelatin; Abcam, Cambridge, UK) or with goat anti-green fluorescent protein (GFP; diluted 1:1000 in PBS/gelatin; Abcam). Secondary antibodies were Alexa 594 goat anti-mouse, for the detection of MAP-2, Alexa 488 goat anti-rabbit (1:1000), for the detection of Tau and Alexa 488-donkey anti-goat (diluted 1:500; Molecular Probes), for the detection of GFP. Cell nuclei were stained with DAPI.

### MORPHOMETRIC ANALYSIS

**FIGURE 1:**
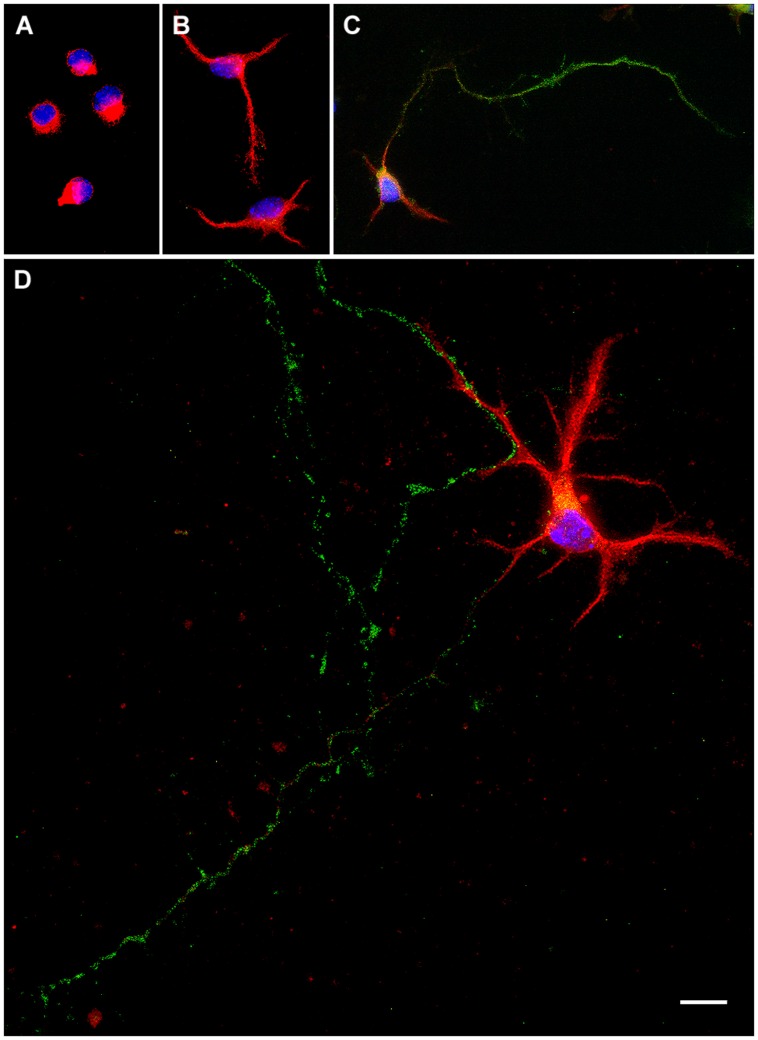
**Representative examples of primary hypothalamic neurons at different stages of development. (A)** Stage I; **(B)** Stage II; **(C)** Stage III and **(D)**, Stage IV. In stage I neurites are not still emerged. Neurons in stage II show short neurites or minor processes. In stage III neurons show a thin, long and Tau-positive neurite, relatively uniform in diameter, which corresponds to the axon. In stage IV dendrites show Y angles branching. Scale bar, 10 μm.

After plating, dissociated neurons undergo several intermediate stages of development (Dotti et al., 1988). The maturation of hypothalamic neurons in cultures can be divided in five morphological stages (Díaz et al., 1992). In stage I cells have a motile lamellipodia around the periphery and neurites are not still emerged (**Figure 1A**). After few hours, in stage II, the lamellipodia condenses at several discrete sites where short neurites or minor processes appear (**Figure 1B**). Stage III is characterized by the formation and growth of the axon, which is a thin, long, and Tau-positive neurite, relatively uniform in diameter (**Figure 1C**). At this stage the neuron turn out to be polarized. Over time, the remaining minor processes begin to elongate, but at a much slower rate than the axon, and acquire the tapering and Y angles branching, characteristic of dendrites at stage IV (**Figure 1D**). Further progress, at stage V, includes the maturation of the axonal and dendritic arbor, and the development of synaptic contacts and dendritic spines. Under our culture conditions, at 7 DIV, all neurons were at stage III or stage IV. Stage V was not observed. In this study we evaluated the proportion of neurons in stages I, II, III, and IV of differentiation from 1 to 7 DIV. In addition, we assessed the number of primary neurites per neuron; the length of minor processes, dendrites, and axon and the proportion of cells with branched neurites. Dendrites were identified as MAP-2 immunoreactive neurites with ramifications in Y angles with shorter, straight segments. The axon was identified as one thin neurite, longer than the others, which is immunoreactive for Tau and not for MAP-2.

The morphometric analysis of immunostained neuronal cultures was performed on digitalized images using the ImageJ 1.38 (NIH). Images were acquired through standard epifluorescence in a Leica microscope equipped with a Leica digital camera (Leica, Heidelberg, Germany) at 20× or 40× magnifications. Images were coded, and the person conducting the analysis was blind to the experimental group. For each sex, time *in vitro* and treatment, 60–80 immunostained neurons that could be identified as individual cells were analyzed; at least four separate cultures were made in each condition.

### STATISTICAL ANALYSIS

Data are expressed by position parameters as media, median, percentiles, and dispersion parameters as SEM and minimum/maximum values. Kruskall Wallis or Mann Whitney-U non-parametric tests for unpaired samples were used to compare the number of neurons (median). According to data distribution, one-way or two-way ANOVA followed by comparison of means by Fisher’s least significance difference (LSD) *post hoc* was used as parametric test. A level of *p* < 0.05 was considered as statistically significant. The n for statistical analysis was the number of independent cultures.

## RESULTS

### SEX DIFFERENCES IN THE MORPHOLOGICAL STAGES OF MATURATION

**FIGURE 2:**
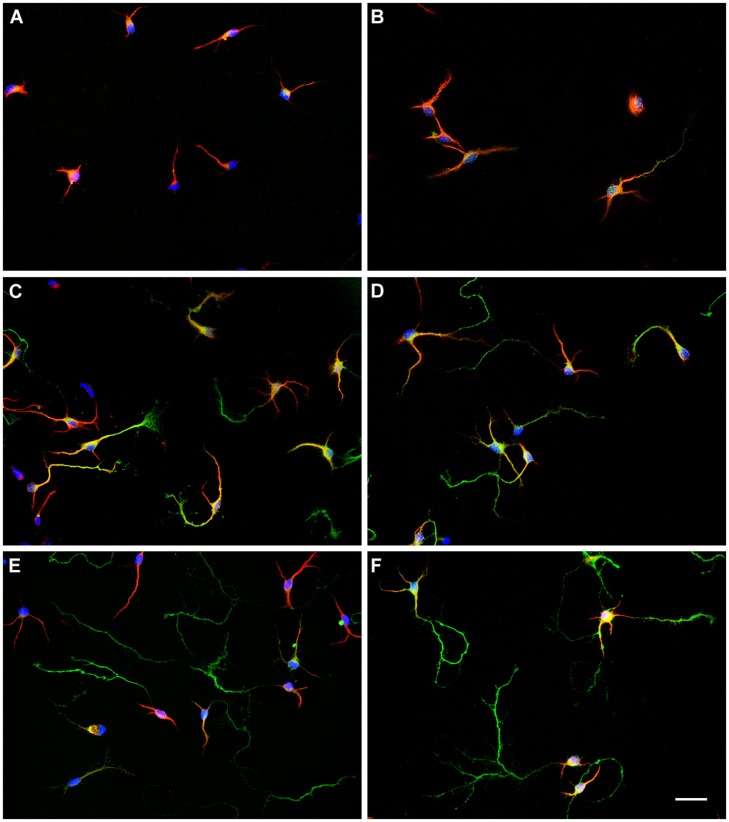
**Representative examples of hypothalamic neurons from male and female embryos at different days *in vitro* (DIV). (A)** 2 DIV, male; **(B)** 2 DIV, female; **(C)** 4 DIV, male; **(D)** 4 DIV, female; **(E)** 6 DIV, male; **(F)** 6 DIV, female. Scale bar, 20 μm.

**FIGURE 3:**
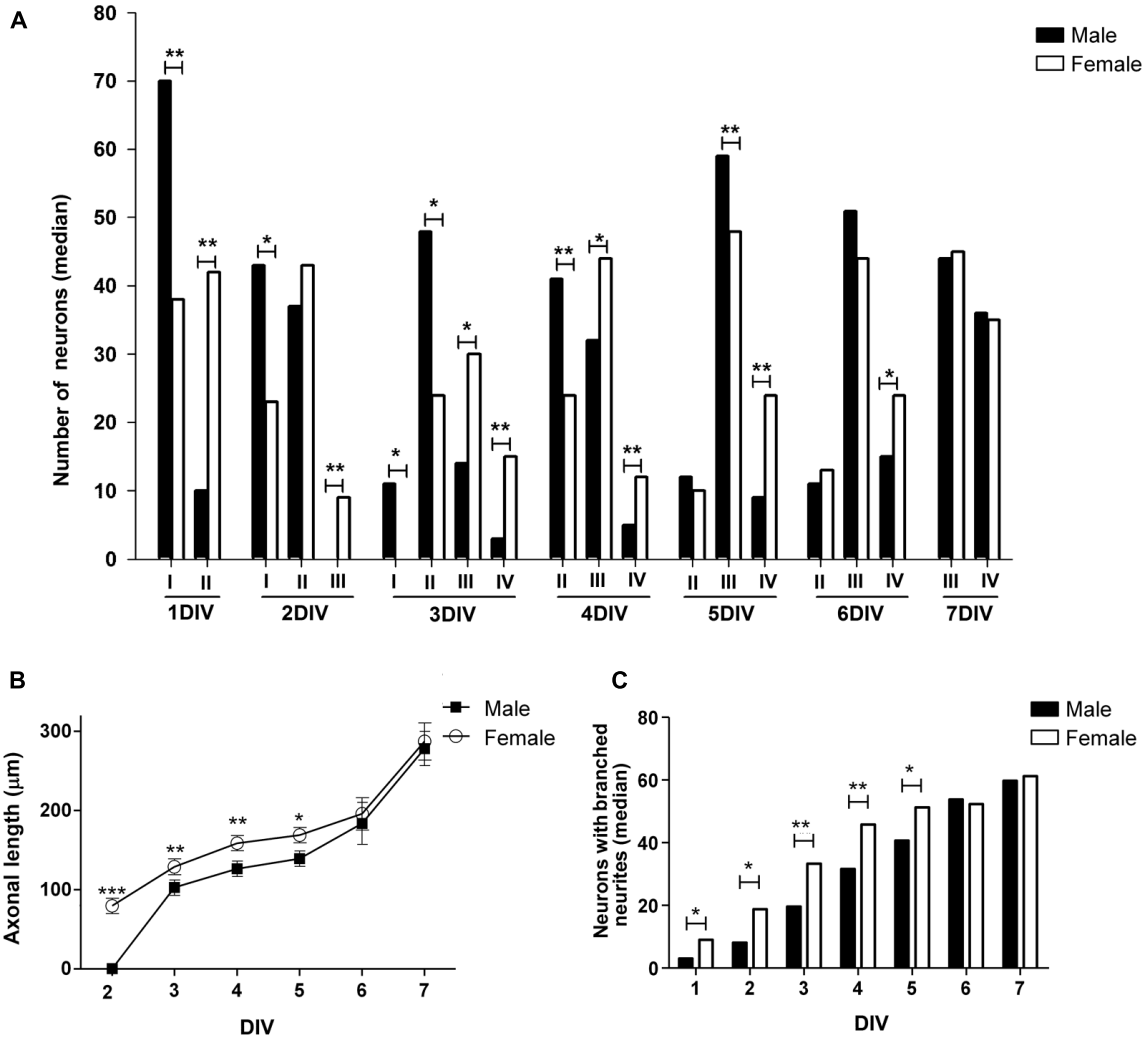
**Changes over time in morphological parameters of hypothalamic neurons in sexually segregated cultures maintained from 1–7 days *in vitro* (DIV). (A)** Median number of neurons at different stage of maturation: I, II, III, and IV (for references see caption in **Figure 1**) **(B)** Mean of axonal length. **(C)** Median number of neurons with branched neurites. Data are expressed as median or mean ± SEM. *n* = 5–6 independent cultures for each sex. *p < 0.050, **p < 0.010, and ***p < 0.001.

To identify sex differences in neuronal development independent of gonadal hormones, hypothalamic neuronal cultures were prepared from male and female E14 mouse embryos, before the peak of testosterone production by fetal testis, which in mice is at E17-18 (O’Shaughnessy et al., 1998, 2006). We analyzed the number of neurons at different stages of maturation from 1 to 7 DIV in sexually segregated cultures (**Figures 2** and 3A). The proportion of cells in early stages of differentiation (I, II) progressively decreased with time in culture, as the proportion of cells in more differentiated stages (III, IV) increased. At 1 DIV neurons were either in morphological stage I or in the morphological stage II of development (**Figure 3A**). Most neurons derived from male remained at the morphological stage I of development compared with female cultures (*p* = 0.008). In contrast, the majority of the neurons derived from female embryos were already in stage II (*p* = 0.008). At 2 DIV cells were in stages I, II, or III of development (**Figures 2A,B** and **3A**). The median number of cells in stage I was still higher in male cultures compared to female cultures (*p* = 0.032), although male and female cultures reached a similar proportion of neurons in stage II. In addition, only some female neurons had clearly differentiated their axons and were, therefore, in stage III of development (sex difference, *p* = 0.008). At 3 DIV cells were in stages I, II, III, or IV (**Figure 3A**). No cells in stage I were detected in female cultures at 3 DIV (sex difference, *p* = 0.047). In addition, female cultures showed a significant decrease in the number of cells at stage II compared to male cultures (*p* = 0.024) and a significant increase in the number of cells at stages III (*p* = 0.016) and IV (*p* = 0.008) compared to male cultures. At 4 DIV cells in stage I of development were no longer observed: cells were in stages II, III, or IV (**Figures 2C,D** and 3A). The proportion of female cells at stage II was significantly decreased compared to male cultures (*p* = 0.008). In contrast, the proportion of cells at stages III (*p* = 0.015) and IV (*p* = 0.008) was higher in female cultures. At 5 DIV neurons were in stages II, III, and IV (**Figure 3A**). Male and female cultures showed similar proportions of cells at stage II. However, female cultures had a reduced proportion of cells at stage III (*p* = 0.008) and an increased proportion of cells at stage IV compared to male cultures (*p* = 0.008). At 6 DIV neurons were in stages II, III, or IV (**Figures 2E,F** and **3A**). Male and female cultures showed a similar number of cells in stage II and in stage III. However, female cultures showed a significant increase in the number of cells at stage IV compared to male cultures (*p* = 0.047). By 7 DIV, cells in male and female cultures were at stages III or IV, with similar number of the cells in each stage for both sexes (**Figure 3A**).

Taken altogether these results indicate that the dynamic of neuronal maturation is different in male and female hypothalamic cultures: female neurons differentiated faster than male neurons in culture until 6–7 DIV when neurons of both sexes acquire the fully differentiated appearance characterized by the common features of dendrites and axons.

### SEX DIFFERENCES IN NEURITE OUTGROWTH

To further obtain detailed information about the described sex differences in neuronal maturation we analyzed different morphological parameters of cellular shape in hypothalamic neurons maintained in culture from 1 to 7 DIV. There were no sex differences in the mean number of primary neurites, whereas there were an effect of time *in vitro* (main effect DIV: *F*_6,51_ = 24.61, *p* < 0.001) as the number of primary neurites increased up to 3 DIV and remained stable thereafter (**Table 1**). The statistical analysis of the length of minor processes (**Table 1**) and the length of dendrites (**Table 1**) indicated a significant effect due to sex (*F*_1,52_ = 8.58, *p* = 0.005 and *F*_1,38_ = 4.34, *p* = 0.044, respectively) and DIV (*F*_6,52_ = 63.43, *p* < 0.001, and *F*_1,4_ = 36.19, *p* < 0.001, respectively), showing a progressive increase in neuritic length in both male and female cultures with time *in vitro*. Regarding to the length of axons a significant effect due to sex (*F*_1,44_ = 878.68, *p* < 0.001), DIV (*F*_5,44_ = 1140.17, *p* < 0.001) as well as a significant interaction of sex and DIV (*F*_5,44_ = 611.77, *p* < 0.001) were found. Fisher *post hoc* analysis revealed that at 2 DIV only female neurons had already extended an axon and, day-by-day comparison, revealed that female neurons showed longer axons than male neurons from 3 to 5 DIV (*p* < 0.05, **Figure 3B**). Moreover, the proportion of neurons with branched neurites was also different in male and female cultures. As shown in **Figure 3C**, female cultures had significantly higher median number of neurons with branched neurites than male cultures from 1 to 5 DIV (*p* ≤ 0.050).

**Table 1:** Number of primary neurites, length of minor processes and length of dendrites in hypothalamic neuronal cultures of male (M) and female (F) embryos.

	Sex	DIV						
		1	2	3	4	5	6	7
**Primary neurites (number)**	M	1.3 ± 0.1^a^	1.5 ± 0.2^b^	2.5 ± 0.3^c^	2.7 ± 0.2^c^	2.2 ± 0.1^c^	2.1 ± 0.1^c^	2.2 ± 0.1^c^
	F	1.3 ± 0.1^a^	1.7 ± 0.2^b^	2.6 ± 0.2^c^	2.7 ± 0.1^c^	2.6 ± 0.2^c^	2.5 ± 0.1^c^	2.3 ± 0.1^c^
**Minor processes (μm)**	M	14.0 ± 1.2^a^	18.8 ± 1.6^b^	28.5 ± 2.6^c^	31.5 ± 2.3^c^	39.8 ± 3.4^d^	47.4 ± 2.3^d^	62.3 ± 3.9^e^
	F	17.8 ± 1.5^f^	23.3 ± 3.3^g^	32.8 ± 2.9^h^	38.8 ± 2.7^h^	43.8 ± 4.0^i^	50.3 ± 3.7^i^	63.2 ± 2.2^j^
**Dendrites (μm)**	M	Absent	Absent	78.4 ± 2.5^a^	94.5 ± 4.2^b^	105 ± 4.7^c^	121.2 ± 11^d^	141.4 ± 16^d^
	F	Absent	Absent	82.8 ± 3.7^e^	103.2 ± 6.9^f^	118.4 ± 6.7^g^	138.3 ± 22^h^	134.8 ± 13^h^

*Data represent the mean ± SEM. *n* = 4–6 independent cultures for each sex. Different letters indicate statistical differences between groups (*p* < 0.05).*

### Ngn3 WAS DIFFERENTIALLY EXPRESSED IN MALE AND FEMALE HYPOTHALAMIC NEURONS

**FIGURE 4:**
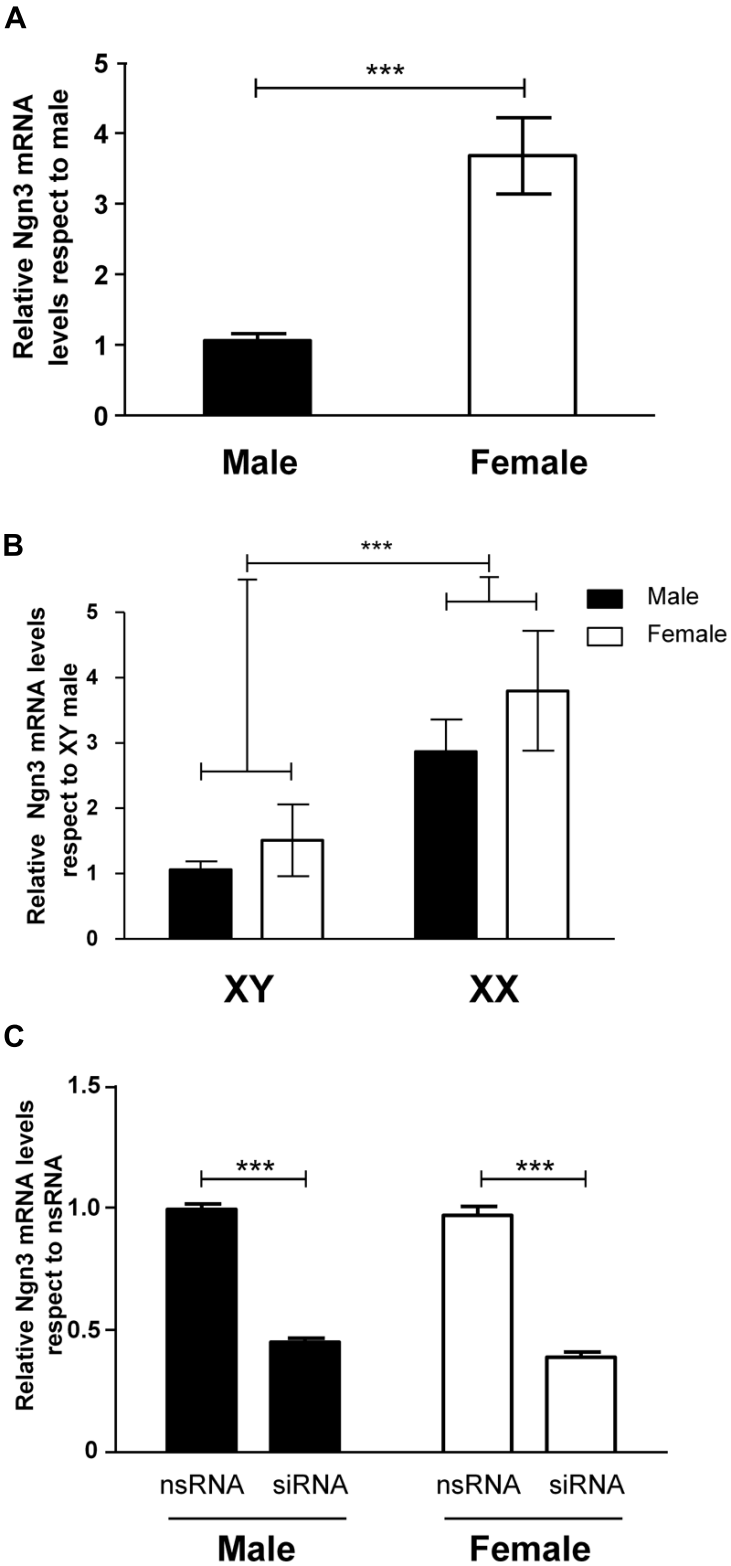
**Sex differences in the expression of Ngn3 in primary hypothalamic neurons. (A)** Ngn3 mRNA levels in male and female hypothalamic neurons at 3 DIV. **(B)** Ngn3 mRNA levels in the four core genotype model. **(C)** Validation of siRNA targeting Ngn3 (siRNA) and a control non-targeting siRNA (nsRNA) in male and female hypothalamic cultures. Data are mean ± SEM. *n* = 6–9 independent cultures for each sex and treatment. LSD test indicated significant differences ***p < 0.001.

Since the transcription factor Ngn3 has been recently involved in the control of neuritogenesis (Salama-Cohen et al., 2006; Ruiz-Palmero et al., 2011), we assessed the expression of Ngn3 in sexually segregated hypothalamic cultures at 3 DIV. The ANOVA analysis revealed a significant effect of sex (*F*_1,14_ = 22.34, *p* < 0.001) in the expression of Ngn3: female cultures showed significantly higher Ngn3 mRNA levels compared to male cultures (*p* < 0.001, **Figure 4A**).

### SEX DIFFERENCES IN Ngn3 EXPRESSION IN PRIMARY HYPOTHALAMIC NEURONS WERE DETERMINED BY SEX CHROMOSOMES

We used the FCG mouse model to determine whether the sex difference in Ngn3 expression in hypothalamic neurons was determined by any sex difference in levels of hormones derived from the gonads at or before E14. Therefore, we analyzed Ngn3 mRNA levels in cultures from XY male/XY female and XX male/XX female mice. Two-way ANOVA revealed no effect of gonadal sex but a significant effect of sex chromosome complement (*F*_1,28_ = 12.60, *p* < 0.001). Neurons carrying the XY chromosomes showed lower expression levels of Ngn3 than neurons carrying XX chromosomes (*p* < 0.001), independently of having been originated from gonadal male or gonadal female embryos (**Figure 4B**). There was no interaction of gonadal sex and chromosome complement.

### DOWN-REGULATION OF Ngn3 EXPRESSION ABOLISHED SEX DIFFERENCES IN MATURATION AND NEURITIC GROWTH OF HYPOTHALAMIC NEURONS

In order to determine whether the observed sex differences in maturation and neuritic growth of hypothalamic neurons depend on the differential Ngn3 expression, we used siRNA strategy to reduce Ngn3 levels. The electroporation delivery of siRNA targeting Ngn3 resulted in a significant decrease in Ngn3 mRNA levels in male and female cultures (*F*_1,8_ = 502.52, *p* < 0.001, **Figure 4C**).

**FIGURE 5:**
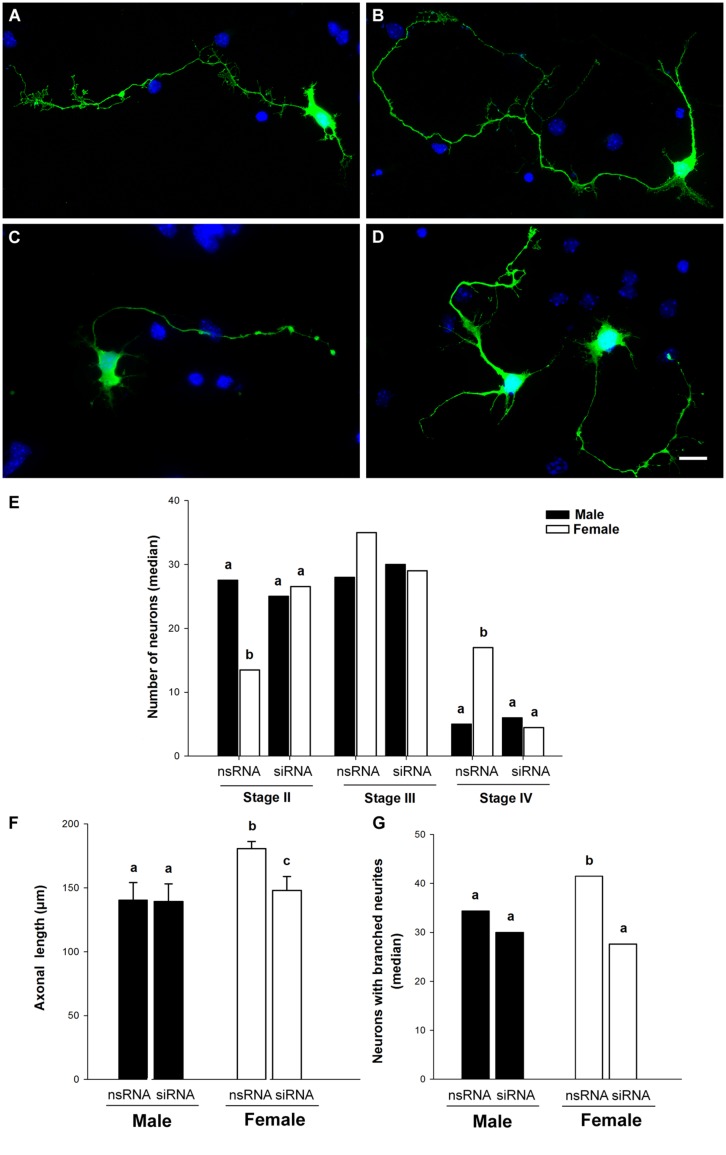
**Down-regulation of Ngn3 expression abolished sex differences in maturation and neuritic growth of hypothalamic neurons.** Neurons were co-transfected with the Ngn3 siRNAs and pEGFP-C2 at 3 DIV and processed for immunostaining 16 h latter. **(A,B)** Representative example of male **(A)** and female **(B)** neurons treated with non-targeting siRNA. **(C,D)** Representative example of male **(C)** and female **(D)** neurons treated with the Ngn3 siRNA. Scale bar, 10 μm. **(E)** Effect of Ngn3 siRNA on the median number of primary hypothalamic neurons at different stages of development. **(F)** Effect of Ngn3 siRNA on the mean of axonal length. **(G)** Effect of Ngn3 siRNA on the median number of neurons with branched neurites. Data are expressed as median or mean ± SEM. *n* = 4–6 independent cultures for each sex and treatment. Different letters indicate statistical differences between groups (*p* < 0.05).

**Figure 5** shows the effects of siRNA targeting Ngn3 on the morphology of hypothalamic neurons of male (**Figures 5A,C,E–G**) and female embryos (**Figures 5B,D,E–G**). Sex differences observed in neuronal differentiation and neuritogenesis under basal conditions were still detected in the cultures transfected with the non-target siRNA (nsRNA). Thus, male cultures treated with the nsRNA showed a significantly higher proportion of cells at stage II of differentiation compared to female cultures (sex difference, *p* = 0.027). In contrast, female cultures showed a higher proportion of neurons at stage IV (*p* = 0.013) of maturation (**Figure 5E**). In addition, male neurons showed shorter axons compared to female neurons (*p* = 0.042; **Figure 5F**) and male cultures showed a decreased proportion of neurons with branched neurites than female cultures (*p* = 0.029; **Figure 5G**). The transfection of siRNA targeting Ngn3 abolished these sex differences, increasing the proportion of female cells at stage II of maturation (*p* = 0.027; **Figure 5E**), decreasing the proportion of female cells at stage IV of maturation (*p* = 0.013; **Figure 5E**), decreasing the length of axons in female neurons (*p* = 0.024; **Figure 5F**) and decreasing the proportion of neurons with branched neurites in female cultures (*p* = 0.029; **Figure 5G**).

In contrast, the Ngn3 siRNA did not have significant effects in the morphology of male neurons (**Figures 5E–G**). The length of minor processes and the length of dendrites were not significantly affected by the transfection of siRNA targeting Ngn3, neither in male nor in female cultures (**Table 2**). The final outcome was that the sex differences in the proportion of cells at different stages of maturation, in axonal length and in neuritic branching, observed under basal conditions, were abolished by the treatment with the Ngn3 siRNA.

**Table 2:** Length of minor processes and length of dendrites in male (M) and female (F) hypothalamic neuronal cultures transfected with siRNA targeting Ngn3 (siRNA) or a control non-sense oligonucleotide (nsRNA).

	Sex	Treatment	
		nsRNA	siRNA
**Minor processes (μm)**	M	34.9 ± 4.0	34.9 ± 3.6
	F	46.7 ± 4.3	36.0 ± 1.9
**Dendrites (μm)**	M	97.8 ± 10.4	107.9 ± 12.0
	F	115.6 ± 9.6	116.5 ± 9.9

*Data represent the mean ± SEM. *n* = 4–6 independent cultures for each sex.*

### ESTRADIOL INCREASED Ngn3 mRNA LEVELS IN MALE, BUT NOT FEMALE, NEURONS

**FIGURE 6:**
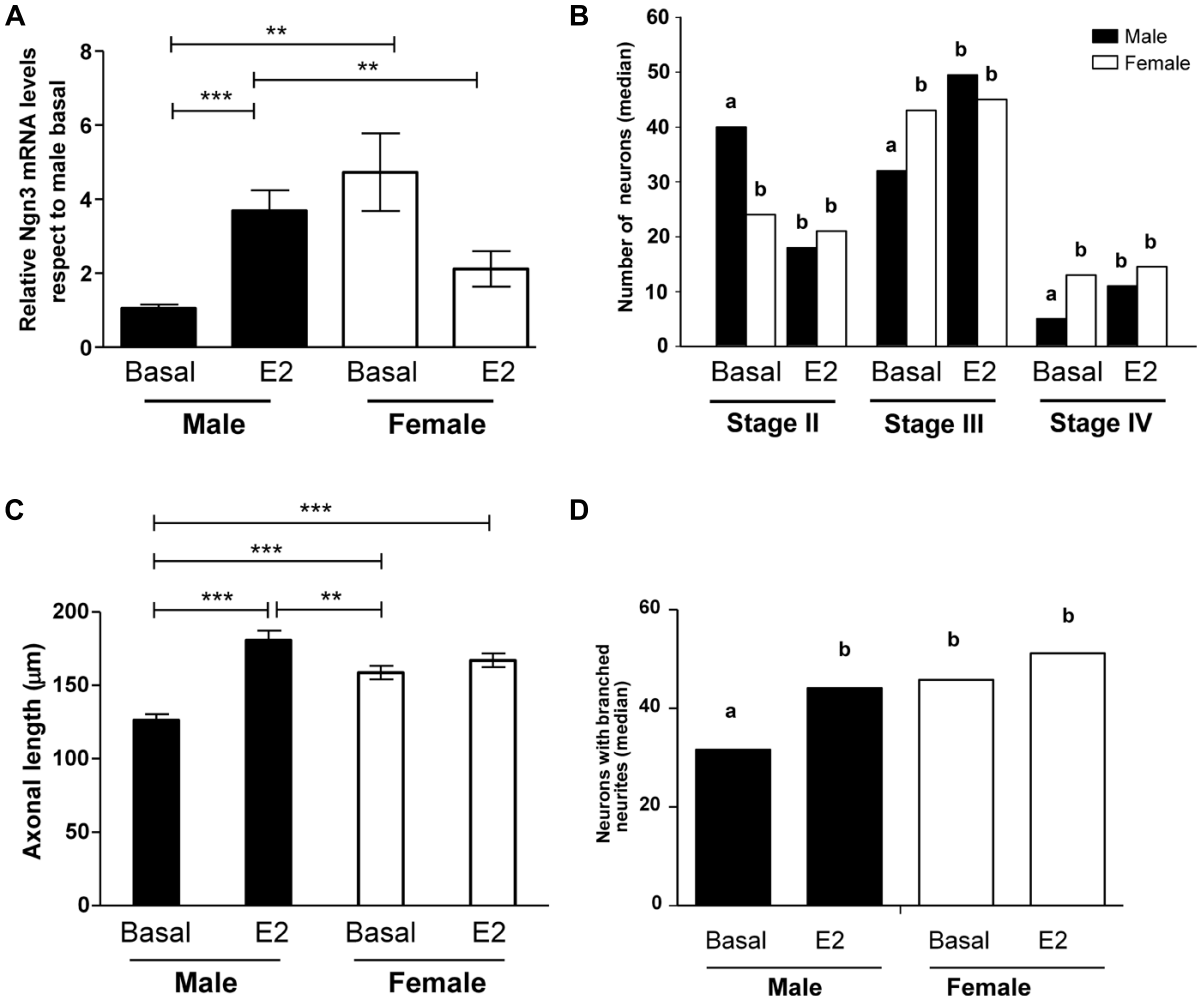
**Effect of 17β-estradiol on Ngn3 mRNA levels and on parameters of neuronal morphology after 4 DIV. (A)** Ngn3 mRNA levels. **(B)** Number of primary hypothalamic neurons at different stages of development. **(C)** Mean of axonal length. **(D)** Median number of neurons with branched neurites. Data are expressed as median or mean ± SEM. *n* = 4–5 independent cultures for each sex and treatment. Different letters indicate statistical differences between groups (*p* < 0.05), **p < 0.010, ***p < 0.001.

Some cultures were incubated for 2 h with 17β-estradiol (10^-10^ M) or vehicle and the levels of Ngn3 mRNA were evaluated. Two-way ANOVA reveled a significant interaction of sex and estradiol treatment (*F*_1,22_ = 17.02; *p* < 0.001). The treatment with estradiol resulted in a significant increase in the expression of Ngn3 in male cultures (*p* < 0.001, **Figure 6A**). Estradiol treatment did not significantly affect Ngn3 mRNA levels in female cultures (*p* = 0.115). Thus, the effect of estradiol in male hypothalamic neurons abolished the sex difference in Ngn3: male cultures treated with estradiol showed similar expression levels of Ngn3 mRNA than female cultures under basal conditions.

### ESTRADIOL ABOLISHED THE SEX DIFFERENCES IN MATURATION AND AXONAL GROWTH OF HYPOTHALAMIC NEURONS

To determine the effect of estradiol on cell morphology, male and female cultures were treated for 4 DIV with the hormone or vehicle. Estradiol promoted the differentiation of male neurons, reducing the proportion of cells in stage II (*p* = 0.008) and increasing the proportion of cells in stages III (*p* = 0.012) and IV (*p* = 0.009; **Figure 6B**). In contrast, estradiol did not significantly affect this parameter in female cultures. The effect of estradiol on male cells resulted in the abolishment of the sex differences in neuronal maturation: male cultures treated with estradiol showed similar proportion of cells at the different stages of development than female cultures under basal conditions or after estradiol treatment.

The analysis of the effect of estradiol treatment on axonal length showed a significant effect of the hormone (*F*_1,14_ = 39.89; *p* < 0.001) as well as a significant interaction of sex with treatment (*F*_1,14_ = 21.35; *p* < 0.001). Estradiol treatment increased axonal length in male neurons (*p* < 0.001, **Figure 6C**). In contrast, estradiol did not significantly affect this parameter in female neurons. The final result was that estradiol treatment reversed the sex difference in axonal length observed under basal conditions. Thus, male neurons treated with estradiol showed longer axons than female neurons under basal conditions (*p* = 0.007).

Estradiol treatment also increased the median number of neurons with branched neurites in male cultures (*p* = 0.015, **Figure 6D**). In contrast, estradiol did not significantly affect this parameter in female neurons. The final result was that estradiol treatment reversed the sex difference in the proportion of neurons with branched neurites.

## DISCUSSION

These results confirm and extent our previous data on the existence of sex differences in the growth and differentiation of hypothalamic neurons before the critical period of brain masculinization. We now also show a sex difference in the expression of the neuritogenic transcription factor Ngn3 that is necessary for the greater development in female cultures. Further, we demonstrate that the sex complement determines the sexually dimorphic expression of Ngn3 since XY vs. XX difference was found irrespective of the gonadal type. These results additionally indicate that E2 added to the culture media abolish all sex difference in the growth and differentiation of hypothalamic neurons at this early developmental age.

Hypothalamic neurons in culture show a progressive transition for different differentiation stages (Díaz et al., 1992), which are similar to those observed in primary hippocampal neurons (Dotti et al., 1988). The proportion of cells in early stages of differentiation (I, II) progressively decreased with time in culture, as the proportion of cells in more differentiated stages (III, IV) increased. The increase in neuronal differentiation with time in culture was accompanied by a progressive increase in the proportion of neurons with branched neurites and by a progressive increase in the length dendrites and axons.

Male and female neurons showed a different rate of differentiation with time in culture. Thus, at 1 DIV, 18% of male neurons showed neurites (Stage II), while 48% of female neurons had already reach this stage. In addition, the proportion of neurons with branched neurites at 1 DIV was higher in female cultures. A similar situation was observed at 2, 3, 4, and 5 DIV, where female neurons showed an enhanced morphological differentiation compared to male neurons. For instance, by 2 DIV, 14% of female neurons were in stage III (polarized neurons), while male neurons had not yet developed an axon. In addition, by 2 DIV, female cultures showed a higher proportion of cells with branched neurites than male cultures. Sex differences in the proportion of neurons at different stages of differentiation were maintained until 4 DIV and sex differences in the proportion of neurons with branched neurites were still observed a 5 DIV. However, by 6 DIV, male and female cultures showed a similar proportion of neurons in stages II, III, and IV and by 7 DIV, all male and female neurons were at stages III and IV, with a similar proportion of cells in each stage in both sexes. These findings suggest that female hypothalamic primary neurons differentiated earlier than male neurons in culture. However, at the end, by 6–7 DIV, male and female neurons reached the same degree of differentiation, indicating that the difference was in the speed of the process and not in the final outcome. Similar findings have been obtained in rat mesencephalic cultures, where the outgrowth of tyrosine hydroxylase-immunoreactive processes initially proceeded at a faster rate in female than in male cultures (Reisert et al., 1989). The biological meaning of transient sex differences in neuronal development is unknown. However, such differences, *in vivo*, could allow the establishment of a sex dimorphic pattern of synaptic connectivity by coordinating the development of pre and postsynaptic structures. According to our findings, female neurons would show a higher number of postsynaptic sites available when early synaptic afferents arrive and could then, by this mechanism, establish a different pattern of synaptic connectivity than male neurons.

Although female neurons were more advanced than male neurons in the development of some morphological characteristics, such as the growth and elongation of the axon and the branching of neurites, this was not the case for other parameters analyzed. Thus, the number of primary neurites per neuron, the length of minor processes and the length of dendrites showed similar values in male and female cultures under basal conditions. Therefore, some parameters of the neuritogenic process are sexually dimorphic and others are sex-independent.

Our findings have revealed a sex dimorphic expression of Ngn3 in hypothalamic neurons. Male neurons, which show a retarded neuritic differentiation compared to female neurons, showed decreased mRNA levels of the neuritogenic factor Ngn3. Given the effect of Ngn3 on neuritogenesis and neuronal differentiation, our findings suggest that the sex differences in Ngn3 expression may be the cause of the sex differences in neuronal maturation. To test this hypothesis we used a Ngn3 siRNA that was previously shown to reduce Ngn3 levels in neurons (Ruiz-Palmero et al., 2011). The Ngn3 siRNA decreased Ngn3 expression with the same efficacy in male and female neurons. However, the Ngn3 siRNA showed morphological effects in female neurons but not in male neurons. Thus, Ngn3 siRNA decreased neuronal maturation, the length of the axon and the proportion of cells with branched neurites in female cultures, but not in male cultures. The final result was that female neurons treated with the Ngn3 siRNA showed similar developmental characteristics than male neurons. Interestingly, the developmental effects of Ngn3 siRNA were restricted to those parameters that showed sex differences. Thus, Ngn3 siRNA did not affect the length of minor processes or the length of dendrites. These findings indicate that the differential expression of Ngn3 mediates sex differences in some specific morphological parameters during neuronal development. Further studies are necessary to determine whether these observations reflect *in vivo* phenomena of embryonic hypothalamic neurons. The limited data available indicates that Ngn3 is expressed in the hypothalamic regions of mouse embryos from E9.5 to E17.5 (Pelling et al., 2011).

To determine whether sex differences in Ngn3 expression in hypothalamic neurons was depending on hormones derived from the gonads at or before E14, we analyzed Ngn3 mRNA levels in hypothalamic neurons originated from XY male vs. XY female, and XX male vs. XX female mouse embryos. No sex differences were found in cultures from embryos with same sex chromosome complement and different gonadal phenotype, indicating that there would not be gonadal hormone effects on the Ngn3 expression in the hypothalamus at this age. On the contrary, our findings indicate that sex chromosome complement determines sex differences in Ngn3 expression: cells carrying different sex chromosome gene(s) showed different Ngn3 mRNA levels (XY < XX). Thus, sex differences in the expression of the autosomal Ngn3 gene should be the consequence of differences in the expression of X or Y chromosome genes that result from the inherent sex difference in the number (two copies of X) and/or type (presence or absence of Y) of sex chromosomes. Therefore, our study has identified a gene, Ngn3, which mediates cell-autonomous actions of sex chromosomes in the generation of sex differences in neuronal development. Additional studies are necessary to find the primary sex chromosome sex-determining gene(s) responsible to activate downstream pathways that differently regulate Ngn3 expression in males and females. Several X/Y-linked genes encoding transcriptional regulator proteins have the potential to mediate chromatin changes and regulate autosomal gene expression (Wijchers and Festenstein, 2011). Sry gene in the Y chromosome, which induces testicular differentiation, can be excluded as a potential candidate for generating this sex difference since XX vs. XY difference in Ngn3 expression was found irrespective of the presence or absence of this gene. Some genes in the X chromosome, such as Kdm6a and Kdm5c (also known as Smcx or Jarid1c), which escape X-inactivation (Reinius et al., 2010; Yang et al., 2010), encode histone demethylases and show higher expression in 2× mice compare to 1× for both sexes (Xu et al., 2002) before and after gonadal differentiation (Wolstenholme et al., 2013). Interestingly, studies in primary mammalian neurons demonstrated a role for Kdm5c in neuritic development (Iwase et al., 2007).

In addition to Ngn3, other genes might be involved in sex chromosome-mediated induction of sex differences in neuronal development. Studies on gene profiling revealed a number of autosomal genes differentially expressed between the sexes and in mice with different number of X chromosomes. Among them, Nptx2, Nedd9, Rorb, Cux2, and Htr3a are involved in neuronal development or associated with neurological diseases (Wolstenholme et al., 2013). Cux2 (cut-like homeobox 2 gene) regulates fundamental aspects of late neuronal differentiation and controls intrinsic mechanisms of dendrite development, spine formation and synaptic function in layers II–III of the cortex (Cubelos et al., 2010). Cux2 has been identified as upstream and downstream target of Notch signaling (Iulianella et al., 2009; Wittmann et al., 2014) and Cux2 overexpression has been shown to inhibit Notch signaling in the olfactory epithelium (Wittmann et al., 2014). This is relevant for our findings, since Ngn3 transcription is negatively regulated by Notch signaling (Salama-Cohen et al., 2006). Whether Cux2 regulates Notch/Ngn3 signaling during neuritogenesis in hypothalamic neurons remains to be determined.

The enhanced differentiation of female hypothalamic neurons observed in our study is in apparent contradiction with the fact that, in general, male neurons in the ventromedial hypothalamus and preoptic area have longer dendrites, increased dendritic surface and more synaptic contacts than female neurons (Ayoub et al., 1983; Matsumoto and Arai, 1986; Madeira et al., 1999; Griffin and Flanagan-Cato, 2009; Flanagan-Cato, 2011). A possible interpretation is that what we have observed in our cultures is the process of neuronal differentiation before the peak of testosterone production by fetal testis. Interestingly, in hypothalamic cultures obtained from E19 rat embryos (i.e., during the peak of fetal testosterone that in rats is on fetal days 18.5–19.5; Huhtaniemi, 1994; Scott et al., 2009), male hypothalamic neurons differentiate axons earlier and have more primary neurites and longer dendrites than female neurons (Díaz et al., 1992). Therefore, based on these previous studies and on our present findings, it may be postulated that female neurons are programmed by sex chromosomes for a faster development than male neurons. However, fetal testosterone, after its intracerebral conversion to estradiol (Naftolin et al., 1972; Zwain and Yen, 1999; Hojo et al., 2004), would reprogram male hypothalamic neurons for an enhanced neuronal differentiation. In this regard, it is interesting to note that in cultures from ventromedial hypothalamus of rat fetuses at E16, only neurons from males respond with increased axonal growth to the addition of estradiol to the culture medium (Cambiasso et al., 1995, 2000). Our present findings confirm this previous observation, showing that estradiol enhanced neuronal differentiation and increased axonal length and the proportion of neurons with branched neurites only in male neurons. The effect of estradiol resulted in the abolishment of the sex differences in the morphological stages of maturation and in the reversion of sex differences in axonal length and the proportion of neurons with branched neurites. This suggests that before the peak of fetal testosterone, male hypothalamic neurons show a slower development but are more sensitive to estradiol than female neurons. This enhanced sensitivity to estradiol in male neurons may facilitate the generation of sex differences after the peak of testosterone production by fetal testis. Interestingly, recent studies have shown that estradiol promotes neuritogenesis by the upregulation of Ngn3 (Ruiz-Palmero et al., 2011). Indeed, the knockdown of Ngn3 expression in primary neuronal cultures using a small interference RNA blocks the effects of estradiol on neuritogenesis, indicating that Ngn3 mediates the action of estradiol on neuritogenesis (Ruiz-Palmero et al., 2011). Our present findings, showing that estradiol increases neuritogenesis in male neurons in parallel with an increase in Ngn3 mRNA levels, suggest that Ngn3 may be crucial for determining not only cell-autonomous actions of sex chromosomes but also hormonal influences on neuronal differentiation.

In summary, we have identified an autosomal gene that mediates cell-autonomous actions of sex chromosomes on the generation of sex differences in neuronal development. Our findings suggest that sex chromosomes enhance female neuronal differentiation by the upregulation of Ngn3. In addition, by maintaining a reduced expression of Ngn3 in male neurons, sex chromosomes may contribute to provide these neurons with an increased responsiveness to estradiol, facilitating the neuritogenic action of the hormone through the upregulation of Ngn3.

## AUTHOR CONTRIBUTIONS

Conceived and designed the experiments: María J. Scerbo, Maria A. Arevalo, Luis M. Garcia-Segura, María J. Cambiasso. Performed the experiments: María J. Scerbo, Alejandra Freire-Regatillo, Carla D. Cisternas. Analyzed the data: María J. Scerbo, Carla D. Cisternas, María J. Cambiasso. Statistical analysis: Mabel Brunotto, María J. Cambiasso. Wrote the paper: Luis M. Garcia-Segura, María J. Cambiasso. Edited the manuscript and helped with the outline of the paper: María J. Scerbo, Maria A. Arevalo.

## Conflict of Interest Statement

The authors declare that the research was conducted in the absence of any commercial or financial relationships that could be construed as a potential conflict of interest.
